# The eleventh hour to enforce rigorous primary cancer prevention

**DOI:** 10.1002/1878-0261.12927

**Published:** 2021-03-04

**Authors:** Joachim Schüz, Carolina Espina

**Affiliations:** ^1^ International Agency for Research on Cancer (IARC/WHO) Lyon France

The numbers of cancer cases are rising dramatically, and no country is an exception to that trend. The Global Cancer Observatory of the International Agency for Research on Cancer/World Health Organization (IARC/WHO) projects an increase of 55% in the annual number of newly diagnosed cancer patients worldwide over the next 20 years, from 18.1 million in 2020 to 28.0 million in 2040, for all cancer sites combined, excluding nonmelanoma skin cancer [1, 2]. This corresponds to a total of more than 450 million new cancer patients over the next 20 years. The projected relative increase is largest (89%) in Africa, the continent with the lowest Human Development Index levels. However, the smaller relative increase of 19% in Europe still means that Europe will remain the continent with the highest number of cancer patients per population size, that is 4.8 million new cases in a population of approximately 730 million projected for 2040 [1]. Globally, mortality from cancer is also rising and is projected to reach 16.2 million in 2040. In 2020, about 27 000 cancer deaths were recorded worldwide every single day [1].

As many have stated before, these massive numbers of cancer patients not only will overburden the healthcare systems of less affluent countries, but also lead to the conclusion that ‘no country can afford to treat its way out of the cancer problem’ [3]. There are also other saddening observations. First, although cancer risk increases steeply with age, cancer affects people of all ages. In 2020, almost one third of the new cancer patients, that is 9.1 million of the 28.0 million, were younger than 65 years when diagnosed with cancer [1]. Second, there are significant social disparities, both within and between countries, in whether a cancer diagnosis is a death sentence. For example, as recently reported for breast cancer, which is the most common cancer type in women, survival of at least 3 years was > 90% in high‐income countries compared with < 40–50% in many sub‐Saharan African countries, and within Namibia, for instance, was 90% in White women compared with < 60% in Black women [4]. Third, and perhaps most saddening, is the fact that all this happens although there is convincing scientific evidence that a large proportion of these cancer cases and deaths are preventable. For instance, the preventable fraction is estimated to be about 40% in high‐income countries [5], including those in Europe [6]. This underlines the need for more rigorous primary prevention of cancer. A long time is needed for cancer prevention interventions to reverse the increasing trends, because of the slow development of the disease. Therefore, the eleventh hour to implement known effective prevention measures is now.

However, the preventive potential differs greatly by cancer type. A disheartening example seems to be lung cancer, which is by far the leading cause of cancer death (about 18% of the total cancer deaths in 2020). The major cause of lung cancer, tobacco smoking, has been known for more than seven decades. Despite this, the epidemic of smoking‐related lung cancer is not under control. Instead, the annual number of new lung cancer cases is projected to increase by 59% over the next 20 years [1]. This relative increase is no different from that of cancer overall or from that of many cancer types for which the causes have not been identified. Nevertheless, there are sparks of hope for a turning point, as shown in more detail in this Special Issue: Peruga *et al*. [7] describe some initial successes of the WHO Framework Convention on Tobacco Control and call for bolder action in, for instance, tobacco taxes and banning certain ingredients in tobacco products. Lung cancer is also the most common occupationally related cancer. In this Special Issue, Olsson and Kromhout outline the preventive potential of rigorously implemented worker protection and reduction of workplace exposures [8]. Yet, another significant risk factor for lung cancer is air pollution. In this Special Issue, Vineis *et al*. [9] describe how cancer prevention could go hand in hand with climate change mitigation on tobacco control, food production and transportation exhaust, creating an effective win–win for people and the environment. Another preventable cancer type, cervical cancer, was responsible for about 600 000 new cancer cases and about 340 000 cancer deaths in 2020 [1]. Cervical cancer can be prevented by a combination of human papillomavirus vaccination and screening, and there is much optimism about the WHO global strategy to eliminate cervical cancer as a public health problem [10]. In this Special Issue, Bigaard and Franceschi [11] describe that even when prevention has overcome the barriers of identifying the causes of a cancer type and successful prevention interventions, some barriers to implementation may remain, such as creating the necessary infrastructure or declines in coverage because of vaccination misinformation. Tobacco use remains the main cause of cancer; it is responsible for almost half of all preventable cancer cases [6]. Other major contributors are unhealthy diet [12] and alcohol consumption [13], as well as obesity, physical inactivity and sedentary behaviour, which are reviewed in detail in this Special Issue by Friedenreich *et al*. [14].

Because smoking, unhealthy diet, obesity, alcohol consumption and physical inactivity are the main contributors to the cancer burden, in the past cancer prevention was too often regarded as an individual’s choice. In this Special Issue, Martin‐Moreno *et al*. [15] describe how individual behaviour is conditioned by environmental factors that ease or impede healthy behavioural choices, and why it is important to opt for multilevel cancer prevention strategies. The assumed costliness of cancer prevention strategies has in the past been another barrier hampering implementation. In this Special Issue, interventions targeting harmful alcohol consumption are used as an example by Cheatley *et al*. [13] to illustrate how to guide health decision‐makers in efficiently allocating resources to meet prevention goals. Finally, in this Special Issue, Puska [16] tackles the so‐called ‘implementation gap’ between cancer prevention recommendations and their application and adherence, including individual behaviour, health service structures and political actions, for the future planning of successful cancer prevention strategies.

The current COVID‐19 crisis highlights the importance of prevention, and this momentum should also be used for cancer prevention. First, authoritative sources of cancer prevention and its successful implementation need to be strengthened to use the best scientific evidence base for cancer control. The European Code against Cancer has proven to be such a key cancer prevention tool [17], and its scale‐up to a region‐adapted World Code against Cancer [18] has been recommended and is now commencing in Latin America and the Caribbean as a second major world region [19]. Second, established effective prevention strategies need to be launched immediately, accompanied by prevention research for interventions not yet scaled up to the population level, for causes of cancer for which the most successful interventions have not yet been identified, and with continued aetiological research aimed at identifying the causes of cancer for the half of the cancer burden for which the causes are still unknown [6, 20]. Third, the COVID‐19 crisis has shown that people can be convinced to follow rigorous measures if the benefits are well explained. Therefore, this may be the right time to more forcefully cut back on smoking and alcohol consumption and to use synergies with essential measures for other noncommunicable diseases or for protection of the environment. For example, reduction in meat consumption reduces the cancer burden, reduces the burden of other major diseases such as cardiovascular disease and diabetes, mitigates climate change, reduces cruelty to animals and reduces inequities in access to food. This Special Issue gives an important overview and perspectives of successful primary cancer prevention.

## Conflict of interest

None declared. Where authors are identified as personnel of the International Agency for Research on Cancer/World Health Organization, the authors alone are responsible for the views expressed in this article, and they do not necessarily represent the decisions, policy, or views of the International Agency for Research on Cancer/World Health Organization.

## Author contributions

All authors contributed to the preparation of the manuscript.
